# Catheter for Hemodialysis in Persistent Left Superior Vena Cava in a Patient with Aortic Valve Endocarditis

**DOI:** 10.21470/1678-9741-2023-0266

**Published:** 2025-03-24

**Authors:** Dejan Marković, Sonja Grković, Vladimir Tutuš, Emilija Nestorović, Duško Terzić, Radmila Karan, Milica Karadžić Kočica, Svetozar Putnik

**Affiliations:** 1 Department of Anesthesia, Clinic for Cardiac Surgery, University Clinical Centre of Serbia, Belgrade, Serbia.; 2 Medical School, University of Belgrade, Belgrade, Serbia; 3 Department of Cardiology, Clinic for Cardiac Surgery, University Clinical Centre of Serbia, Belgrade, Serbia.; 4 Department of Surgery, Clinic for Cardiac Surgery, University Clinical Centre of Serbia, Belgrade, Serbia.

**Keywords:** Cardiac Surgery, Hemodialysis, Central Venous Catheter, Persistent Left Superior Vena Cava

## Abstract

Persistent left superior vena cava (PLSVC) is a common congenital venous anomaly, usually associated with other congenital heart diseases (12%). Its incidence in the general population is 0.5%. In cardiac surgery patients, it is suspected when using the left subclavian vein or left internal jugular vein for central venous catheter or hemodialysis catheter placement. Transthoracic ultrasound exam is useful in confirming the position of catheters in the venous system by injecting a 5% glucose solution that can be visualized in the right atrium after administration through the catheter. Hemodialysis catheters can be inserted in the PLSVC with good catheter function and no major risk in increase of complications.

Persistent left superior vena cava (PLSVC) is the most common congenital venous anomaly of the thoracic venous system. The incidence of this anomaly occurs in 0.3 to 0.5% of the general population, while in people with congenital heart disease, the incidence is of approximately 12%^[1]^. It is most commonly associated with atrial septal defect, ventricular septal defect, aortic coarctation, tetralogy of Fallot, and anomalous pulmonary venous drainage. One of the most common clinical manifestations of PLSVC is arrhythmia — atrial fibrillation, sinus bradycardia, or sinus arrest. Most PLSVCs are diagnosed with difficulty in placing electrodes during the implantation of a permanent pacemaker or different vascular catheters^[2,3]^.

## CASE PRESENTATION

A 36-year-old male patient was admitted from another hospital as an emergency case due to acute endocarditis of the aortic valve, caused by *Enterococcus*. At admission he was unstable, arterial pressure was 80/50 mmHg, he was arrhythmic (atrial fibrillation), heart rate was 125/min, and he was dyspneic, febrile (39 °C), and oliguric. Estimated ejection fraction (EF) was 15-20%, white blood cells count was 20 × 10³, urea was 20 mmol/L, creatinine was 413 µmol/L, glomerular filtration rate was 14, and the chest X-ray showed massive bilateral pleural effusions and pneumonia of the right lower lobe. He was treated with the triple antibiotic therapy (vancomycin, gentamicin, metronidazole).

## TECHNICAL DESCRIPTION

After a short preparation, the patient was promptly transferred into the operating room. The arterial cannula was placed into the left radial artery. As regards placement of the venous catheters, two attempts of puncturing the right jugular vein were unsuccessful, and on the left, also after several attempts, it was not possible to locate the internal jugular vein, so a central venous catheter (CVC) with two ports and a sheath for Swan-Ganz catheter was placed through the right subclavian vein and due to the presence of oliguria and elevated values of urea and creatinine, the hemodialysis catheter was placed through the left subclavian vein. The confirmation that we punctured the central veins was based on the color of the blood, the retrograde blood flow, and the direct measurement of pressure values by connecting the line for invasive monitoring to the needle in the blood vessel. The surgical intervention has passed without complications. A bicuspid aortic valve was found and replaced with the mechanical aortic valve. About 3000 ml of serous content was released from both pleural spaces also. The patient was separated from the cardiopulmonary bypass with the rhythm of the temporary pacemaker and inotropic support of adrenaline (0.05 mcg/kg/min).

Upon arrival at the intensive care unit, a chest X-ray was performed, and a normal CVC position was observed in the right subclavian vein, but the position of the catheter for hemodialysis was atypical. The top of the catheter for hemodialysis was in the projection of the left heart ventricle. At first, we checked the values of central venous pressure (CVP) from both sides, and they were the same. Also, we did the blood gas analyzes from both catheters, and the results were the same (PaO_2_ 4.1 kPa, PaCO_2_ 5.1 kPa, SpO_2_ 60%), which was the confirmation that the catheter for hemodialysis is in the venous system, since the saturation of the blood from the arterial line was 100%.

**Fig. 1:**
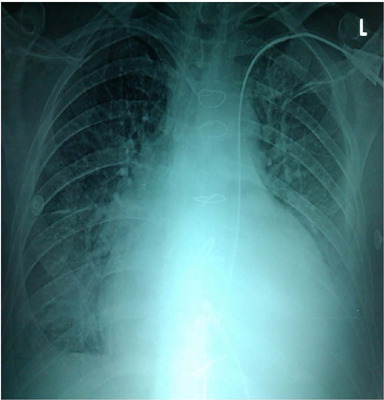
Chest X-ray with contrast trough the hemodialysis catheter in left subclavian vein.

On the first postoperative day, the patient needed the hemodialysis. However, the radiologist gave the contrast and described that the tip of the catheter was located in the projection of the left heart ventricle and that hemodialysis through this catheter is contraindicated (Figure 1).

We performed an ultrasound examination of the heart, which showed that the tip of the catheter is not located in the left ventricle but near the coronary sinus in the right atrium and that it is likely to be a PLSVC. Transthoracic ultrasound examination confirmed the position of catheter for hemodialysis in the venous system by injecting a 5% glucose solution that was visualized in the right atrium after administration through the catheter for hemodialysis. On the suggestion of the ultrasonographer, the catheter was mobilized backwards by 2 cm. After that, the hemodialysis was performed for five days through this catheter without any complications. On the fifth postoperative day, inotropic support was ceased, and the patient was stable and mobilized, he was no longer febrile, the inflammation parameters declined, the International Normalized Ratio was in the therapeutic range, urea and creatinine levels were still elevated (urea 20 mmol/L, creatinine 550 mmol/L), but the urine output was about 2000 ml/24 hours for the previous two days. At the control ultrasound examination of the heart, the EF was approximately 45%, and on the sixth postoperative day the patient was transferred into ward. On the 11^th^ postoperative day, the patient suffered sudden cardiac arrest and was resuscitated, however, a fatal outcome occurred. According to the autopsy findings, there was a thrombosis of periprostatic venous plexus and thromboembolism in the branches of the pulmonary artery, and the conclusion from the autopsy was that the fatal outcome was the result of an acute failure of the right heart due to pulmonary embolism.

## COMMENT

Here we presented a patient with aortic valve endocarditis associated with acute renal failure and the placement of the catheter for hemodialysis in PLSVC. The position of vascular catheters is usually checked by a chest X-ray. In this case, after the radiography, there were two questions.

First, “Is the catheter for hemodialysis in the venous or arterial system or even outside the blood vessels in the mediastinum?”. The second question is “Can the hemodialysis be performed through a catheter in PLSVC?”. Aspiration of blood from both ports of the hemodialysis catheter excluded the possibility of the tip of the catheter be located outside the blood vessels. Although the results of the measured CVP were identical across both catheters, and blood gas analyses from the catheter for hemodialysis undoubtedly indicated that the catheter was in the venous system, yet for the start of hemodialysis another confirmation of the position of the catheter was needed. The position of the catheter can be checked by contrast venography, ultrasound examination of the heart, computerized tomography with venography, or magnetic resonance.

We did the least invasive diagnostic procedure — ultrasound examination of the heart. Transthoracic ultrasound examination confirmed the position of catheter for hemodialysis in the venous system. This finding corresponded to the literature data that the PLSVC is drained to the right atrium in 80-92% of cases, while in the remaining 8-20% it can be drained to the left atrium^[4,5,6]^.

The answer to the question “Can the hemodialysis be performed through this catheter or is it necessary to place the catheter through a vein on the right side?” we got from the available literature data. Based on those experiences, hemodialysis through the catheter in PLSVC is possible, but before starting the dialysis, it is necessary to exclude the existence of the cardiac shunt^[7]^.

In this case, hemodialysis was performed five times without any complications, but on the 11^th^ postoperative day, there was a sudden onset of cardiac arrest and fatal outcome. The reason for the occurrence of a heart failure in this case was not entirely clear.

There have been reports of cases of coronary sinus syndrome, arrhythmia, and sinus arrhythmia in patients with CVC placed in PLSVC, although these complications have been described after giving some drugs through the catheter, rather than as a consequence of the catheter’s presence alone^[8]^.

In patients with congenital heart defects, thoracic venous system anomalies should also be expected, which is important because of the insertion of different vascular catheters. Transthoracic ultrasound examination can be useful in confirming the position of catheters in the venous system by injecting a 5% glucose solution that can be visualized in the right atrium after administration through the catheter. Hemodialysis catheters can be inserted in the PLSVC with good catheter function and no major risk in increase of complications.

## Abbreviations, Acronyms & Symbols

CVC: Central venous catheter
CVP: Central venous pressure
EF: Ejection fraction
PLSVC: Persistent left superior vena cava
